# Exosomal lncRNA-p21 levels may help to distinguish prostate cancer from benign disease

**DOI:** 10.3389/fgene.2015.00168

**Published:** 2015-05-06

**Authors:** Mustafa Işın, Ege Uysaler, Emre Özgür, Hikmet Köseoğlu, Öner Şanlı, Ömer B. Yücel, Uğur Gezer, Nejat Dalay

**Affiliations:** ^1^Department of Basic Oncology, Oncology Institute, Istanbul University, Istanbul, Turkey; ^2^Department of Urology, School of Medicine, Istanbul Hospital, Başkent University, Istanbul, Turkey; ^3^Department of Urology, Istanbul Medical Faculty, Istanbul University, Istanbul, Turkey

**Keywords:** prostate cancer, exosome, lncRNA, benign prostatic hyperplasia, non-invasive diagnosis

## Abstract

Exosomes are membranous vesicles containing various biomolecules including lncRNAs which are involved in cellular communication and are secreted from many cells including cancer cells. In our study, investigated the exosomal GAS5 and lincRNA-p21 lncRNA levels in urine samples from 30 patients with prostate cancer (PCa) and 49 patients with benign prostatic hyperplasia. Quantification of lncRNA molecules was performed by real-time PCR. We observed a significant difference in the exosomal lincRNA-p21 levels between PCa and BPH patients whereas the GAS5 levels did not reveal a difference. Our data suggest that the discriminative potential of exosomal lincRNA-p21 levels may help to improve the diagnostic prediction of the malignant state for patients with PCa.

## Introduction

Prostate cancer (PCa) is the second most common malignancy and accounts for 15% of the cancers in men. It is the fifth leading cause of death and almost 70% of the cases occur in the developed countries (Ferlay et al., 2015). The diagnosis of PCa is performed via histopathological evaluation of biopsy samples which has several known disadvantages like bleeding and infection (Loeb et al., 2013). Although use of the prostate specific antigen (PSA) as a diagnostic marker has improved the detection and management of PCa (Lippi et al., 2009) its low specificity and lack of other predictive parameters for the progression of the disease makes the stratification of the patients with high risk or indolent PCa difficult (Roddam et al., 2005; Nogueira et al., 2010).

Recently, a prostate specific lncRNA, the PCa Gene 3 (PCA3), has been approved as an additional test to determine the need for biopsies in PCa. Unfortunately, negative PCA3 results in indolent cancer carriers and high grade prostatic intraepithelial neoplasia (HGPIN; Morote et al., 2010; Auprich et al., 2011a) renders the biomarker insufficient. The level of TMPRSS2-ERG fusion transcripts, in cells collected from urine after digital examination, have also been evaluated together with the PCA3 levels for a more accurate diagnosis (Leyten et al., 2014). However, none of these approaches can satisfactorily distinguish high-risk from indolent cancer. Therefore identification of new biomarkers to correctly identify patients needing more aggressive treatment would help to prevent individuals with localized tumors from getting unnecessary biopsies and from the side effects of overtreatment.

Exosomes are small membranous vesicles originating from the endosomal compartment which function as messengers in intercellular communication (Simpson et al., 2008; Bang and Thum, 2012; Gezer et al., 2014). They are secreted and released by cells and bind to the receptors on recipient cells, thereby transferring the signal. Exosome secretion is an evolutionary conserved cellular mechanism dating back to Archaea but their role as cellular messengers has been described only recently (Taylor et al., 2006; Deatherage and Cookson, 2012). Exosomes contain proteins and various types of RNA molecules including lncRNAs. Non-exonic transcripts as a whole constitute the majority of non-ribosomal RNA molecules in the cell and lincRNAs constitute a significant portion of this fraction (Kapranov et al., 2010). Exosomes secreted from prostate can be detected in semen (Ronquist and Brody, 1985) and urine (Pisitkun et al., 2004). In recent years it has been shown that PCA3 and several other microRNAs are present in exosomes isolated from PCa patients (Dijkstra et al., 2014; Huang et al., 2015).

LincRNA-p21 and GAS5 lncRNA act as tumor suppressor molecules in the cellular machinery (Schneider et al., 1988; Huarte et al., 2010). Expression of lincRNA-p21 is stimulated by the p53 tumor suppressor protein and upon transcription it suppresses expression of the genes transcriptionally regulated by p53 by binding to the hnRNP-K complex (Huarte et al., 2010). GAS5 plays a role in the induction of apoptosis. It suppresses several antiapoptotic genes by binding to the glucocorticoid receptor (GR) and hence prevents GR from binding to the glucocorticoid response elements on the target DNA molecule (Kino et al., 2010). Recent data indicate that lncRNA molecules may exhibit tissue- and disease-specific expression which can provide important potential biomarkers specific to the particular cancer types (Yaman Agaoglu et al., 2011; Gezer et al., 2014). However, it should be noted that lncRNA molecules are associated both with cancer and pluripotency which can be a confounding factor (St. Laurent et al., 2013). In this study, we aimed to evaluate the diagnostic utility of exosomal lincRNA-p21 and GAS5 levels in individuals with benign prostatic hyperplasia (BPH) and PCa.

## Materials and Methods

### Exosomal RNA Isolation

Thirty patients with PCa (median age 64 ± 6) and 49 patients with BPH (median age 68 ± 9) were enrolled in the study. The study was approved by the Ethics Committee of the Istanbul Faculty of Medicine and informed consent was obtained from the participants. All patients were Caucasian and the disease scores of the patients are given in Table 1. Urine samples from patients were collected after digital rectal examination and were centrifuged for 10 min at 1000 rpm in a conical tube to remove cell and debris. Supernatant was transferred to another conical tube and centrifuged at 2000 rpm for 10 min to remove remaining debris and bacteria. 10 ml of cell-free urine was then transferred to a new 15 ml tube and stored at –80°C until use. Exosomal RNA was extracted according to the manufacturer’s protocol using the “Urine Exosome RNA Isolation Kit” (Norgen Biotek, Thorold, ON, Canada).

**TABLE 1:** **Disease scores of the patients**.

**Benign prostatic hyperplasia patients**	***n* = 49**
IPSS Score (Barry et al., 1992)	
0–7	0
8–19	24
20–35	17
Unknown	8
**Prostate cancer patients**	***n* = 30**
Gleason score (Epstein, 2010)	
6 (3+3)	19
7 (3+4)	3
7 (4+3)	4
Unknown	4

### Quantification of lncRNAs

Exosomal RNA isolated from urine samples were used for cDNA synthesis using the First Strand cDNA Synthesis kit (Thermo Scientific, West Palm Beach, FL, USA) according to the manufacturers’ instructions. The real-time amplification of lncRNA molecules was performed using the LightCycler 480 system (Roche, Germany). SYBR Green (Roche) was used as the fluorescent molecule. The primer sequences are shown in Table 2.

**TABLE 2:** **The primer sequences used in the study**.

**Gene**	**Primer sequence**	**Reference**
LincRNA-p21	F 5′- GGGTGGCTCACTCTTCTGGC -3′	Huarte et al. (2010)
	R 5′- TGGCCTTGCCCGGGCTTGTC -3′	
GAS5	F 5′-CTTCTGGGCTCAAGTGATCCT -3′	Khalil et al. (2009)
	R 5′- TTGTGCCATGAGACTCCATCAG -3′	
GAPDH	F 5′-GCTCTCTGCTCCTCCTGTTC -3′	Khalil et al. (2009)
	R 5′- ACGACCAAATCCGTTGACTC-3′	

The PCR reaction included an initial “hot start” for 10 min., followed by 45 cycles of amplification. Each cycle consisted of a denaturation step at 95°C for 10 s, annealing starting at 60°C for 20 s and decreasing by 2°C every two cycles down to 55°C, and amplification at 72°C for 30 s. For quantification of lncRNAs, the ΔΔCt method was used. The GAPDH gene was used as the reference. All experiments were performed twice and the mean values were calculated.

### Statistical Analyses

SPSS^®^ ver.21.0 statistical program was used for statistical analysis. Kruskal–Wallis or Mann–Whitney U tests were used when appropriate to compare the parameters (lncRNA and PSA levels). A *p*-value ≤ 0.05 was considered statistically significant.

## Results

**FIGURE 1:**
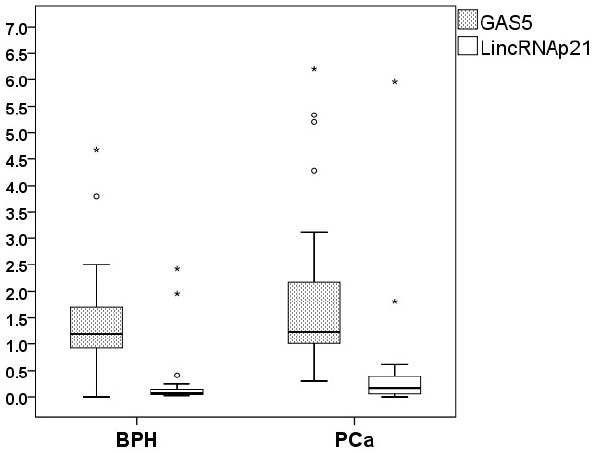
**Distribution of the exosomal GAS5 and lincRNA-p21 levels in the disease groups.** The circles and asterisks represent the values (outliers) falling beyond the statistical distribution of the related variables (exosomal lincRNA-p21 or GAS5).

Exosomal levels of GAS5 and lncRNA-p21 lncRNAs were evaluated in the urine samples of 49 patients diagnosed with BPH and 30 patients with PCa. The distribution of exosomal lncRNA levels are shown in Figure 1. The lincRNA-p21 levels were significantly higher in PCa than in BPH (median; 0.163 vs 0.071; *p* = 0.016, AUC: 0.663). Exosomal GAS5 levels were found to be similar in the two disease groups (median; 1.197 vs 1.235 and *p* = 0.127). The data are shown in Table 3.

**TABLE 3:** **Median values of exosomal lncRNAs and their statistical significances**.

**Median**	**BPH**	**PCa**	**Significance (*p*-value)**
GAS5	1.197	1.235	0.127
LincRNA-p21	0.071	0.163	0.016
AUC value of LincRNA-p21: 0.663, CI:95%			

The PSA levels were higher in the PCa group than in the patients with BPH (*p* < 0.001, median values; 7.7 and 2.16 respectively). In the BPH group no correlation was observed between the IPSS (International Prostate Symptom Score) score and GAS5 or lincRNA-p21 levels. Likewise, no correlation between the clinical stage (Gleason score) and exosomal lncRNA levels was observed in the PCa group. There was no correlation between the PSA levels and the IPSS score in patients with BPH but a correlation was observed between the PSA values and the Gleason score in the PCa group (*p* = 0.123 and 0.049, respectively).

**FIGURE 2:**
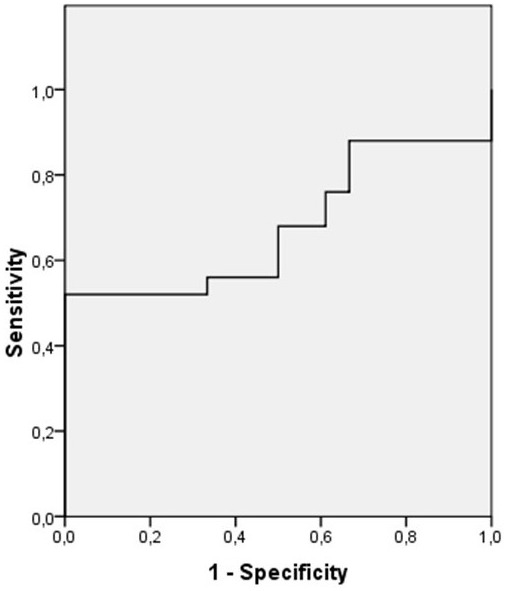
**The ROC curve for the LincRNA-p21 and PSA combination**.

The sensitivity and specificity of lincRNA-p21 and lincRNA-p21 in combination with PSA were calculated using a cut off value of 2.5 ng/ml for PSA and 0.181 for exosomal lincRNA-p21 expression (Figure 2). The specificity for predicting PCa increased from 63 to 94% when the two parameters were combined while the specificity did not change (Table 4).

**TABLE 4:** **Sensitivity and specificity of exosomal lncRNA-p21 and of the lincRNA-p21/PSA combination**.

	**LincRNA-p21**	**LincRNA-p21 and PSA**
Sensitivity	67%	52%
Specificity	63%	94%

## Discussion

Our study is the first report, revealing presence of lincRNA-p21 and GAS5 lncRNA molecules in the exosomes derived from urine samples. Circulating GAS5 and lincRNA-p21 have been previously detected in B-cell malignancies (Isin et al., 2014) but exosomal GAS5 and lincRNA-p21 molecules have previously been only reported in exosomes secreted from HeLa and MCF-7 cell lines but not in human tumors (Gezer et al., 2014). Cellular lincRNA-p21 expression has been suggested to affect global gene expression in different cancers by modulating mRNA translation and supressing the p53 and Wnt/β-catenin signaling pathways (Dimitrova et al., 2014; Wang et al., 2014). Cellular GAS5 expression suggest a role of GAS5 in the regulation of apoptosis in breast cancer cell lines and tumors (Pickard and Williams, 2014) and an inverse association with the mTOR expression in PCa cell lines (Yacqub-Usman et al., 2015). In the present study, we observed significantly higher levels of exosomal lincRNA-p21 levels in the patients with PCa.

It has been shown that one in six of the prostatectomy specimens may contain indolent cancers which usually do not progress to clinical detection during the lifetime of the patient (Epstein et al., 1994). On the other hand, several studies suggest that more than 50% of the cancers which are initially diagnosed as localized tumors are actually advanced at the time of treatment (Chun et al., 2008; Jeldres et al., 2008).

The PSA test is not sensitive enough to predict the presence, extent and risk of recurrence of PCa (Albertsen, 2010; Zeliadt et al., 2010; Friedrich, 2011). Therefore, there is a definite need for non-invasive diagnostic biomarkers which can distinguish the low- and high-risk patients in the clinical decision-making. Even though non-invasive detection of PCA3 levels in urine after digital rectal examination may provide some useful information on the need of a repeated biopsy, evaluation of PCA3 also fails to be specific (Bradley et al., 2013). Dijkstra et al. (2014) have shown that the diagnostic performance of PCA3 in exosomes was found to be more successful than in urine where the PCA3 score was normalized with PSA mRNA in order to achieve a higher performance. Although their study group is quite small the authors suggested an advantage of exosomal PCA3 evaluation while indicating that their data should be validated in larger clinical cohorts. The discriminative capacity achieved by the exosomal PCA3 in this study (AUC: 0.524) is lower than lincRNA-p21 (AUC: 0.663, CI: 95%) in the present study. These data indicate that analysis of exosomal lincRNA-p21 in urine performs better than PCA3 in detecting PCa.

Recently, there is an active controversy over decreasing the cut-off level of PCA3 from 35 to 25 (Nakanishi et al., 2008; Auprich et al., 2011b; de la Taille et al., 2011; Ploussard et al., 2011) which is expected to increase its diagnostic sensitivity. A meta-analysis by Luo et al. (2014) reported that the sensitivity of PCA3 for detecting PCa ranges from 46.9 to 82.3% and specificity from 55 to 92%. In our study the specificity of lincRNA-p21 for PCa was 94% when combined with PSA.

Expression of TMPRSS2-ERG has also been analyzed in urine samples (Leyten et al., 2014). It has been reported that combination of PCA3 and TMPRSS2-ERG expression increased the sensitivity of detecting PCa. However, the biomarker pair still failed to detect indolent tumors with a Gleason score of ≥7.

A new study investigating exosomal microRNA molecules derived from plasma samples in 29 castration resistant PCa (CRPCa) patients reported two significant miRNA molecules (miR-1290 and miR-375) which were later evaluated in 100 patients with CRPCa, as a prognostic marker significantly associated with poor prognosis, needing prospective validation (Huang et al., 2015).

In absence of reliable markers for detection and classification of PCa urinary exosomal lncRNAs may can provide an alternative, and non-invasive source of biomarkers. The stability and longevity of the RNA molecules is ideal for non-invasive diagnosis and characterization of the tumors. Our study for the first time demonstrates that detection of exosomal lncRNAs in urine may act as suitable biomarkers with potential utility of therapeutic implications. LincRNA-p21 provides a promising marker with therapeutic potential for the detection and stratification of PCa. Further studies with larger patient groups are needed to validate the therapeutic utility of exosomal lincRNA-p21 levels in urine.

### Conflict of Interest Statement

The authors declare that the research was conducted in the absence of any commercial or financial relationships that could be construed as a potential conflict of interest.
